# Supplementary material for: Hybridization of short-range and long-range charge transfer excited states in multiple resonance emitter
Source: Nat Commun. 2023 Aug 9;14:4818. doi: 10.1038/s41467-023-40481-w (PMC10412604; doi:10.1038/s41467-023-40481-w)
Supplement: Supplementary file 1 — Supplementary Information [file 41467_2023_40481_MOESM1_ESM.pdf]

Supplementary Information: Hybridization of Short-Range and Long-Range Charge Transfer Excited States in Multiple Resonance Emitter

Ha Lim Lee[1+], Jihoon Kang[1+], Junseop Lim[1], Seung Chan Kim[1], Soon Ok Jeon[2*], Jun Yeob Lee[1,3,4*]

[1]School of Chemical Engineering, Sungkyunkwan University

2066, Seobu-ro, Jangan-gu, Suwon-si, Gyeonggi-do, 16419, Korea

[2]Samsung Advanced Institute of Technology, Samsung Electronics,

130 Samsung-ro, Suwon, Gyeonggi, 16678, Republic of Korea

[3]SKKU Advanced Institute of Nano Technology, Sungkyunkwan University

2066, Seobu-ro, Jangan-gu, Suwon, Gyeonggi, 16419, Republic of Korea

[4]SKKU Institute of Energy Science and Technology, Sungkyunkwan University

2066, Seobu-ro, Jangan-gu, Suwon, Gyeonggi, 16419, Republic of Korea

E-mail: so.jeon@samsung.com, leej17@skku.edu

* To whom correspondence should be addressed

[+] Equal contribution

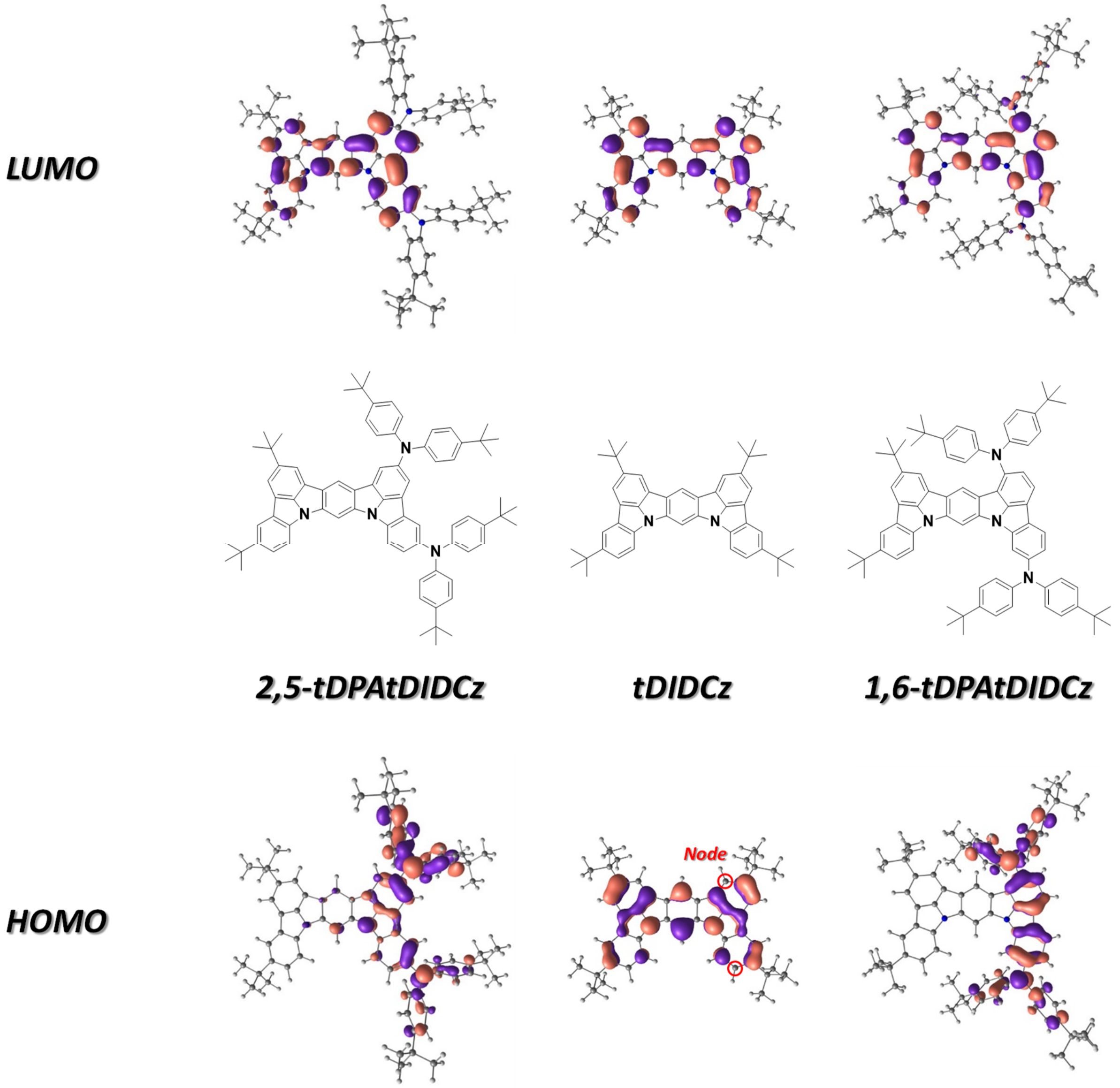

Supplementary Fig. 1 Frontier molecular orbital distributions. The distributions of the highest occupied molecular orbitals (HOMO) and the lowest unoccupied molecular orbitals (LUMO) of 2,5-tDPAtDIDCz (left), tDIDCz (mid) and 1,6-tDPAtDIDCz (right). The nodes of HOMO distribution of tDIDCz are marked in red circle.

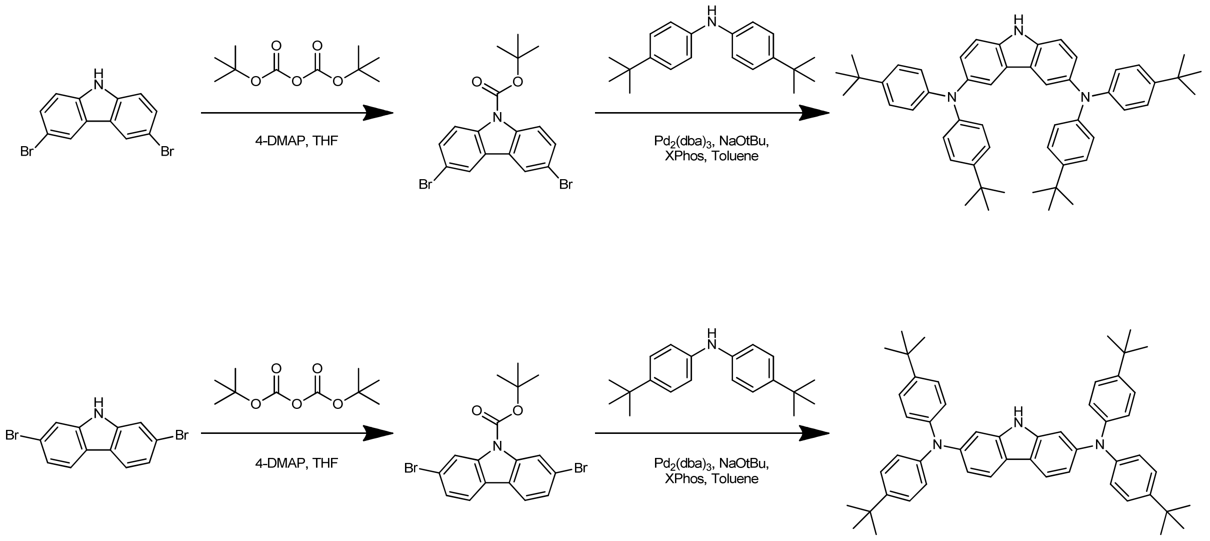

Supplementary Fig. 2 Synthesis. Synthetic scheme of intermediate donors.

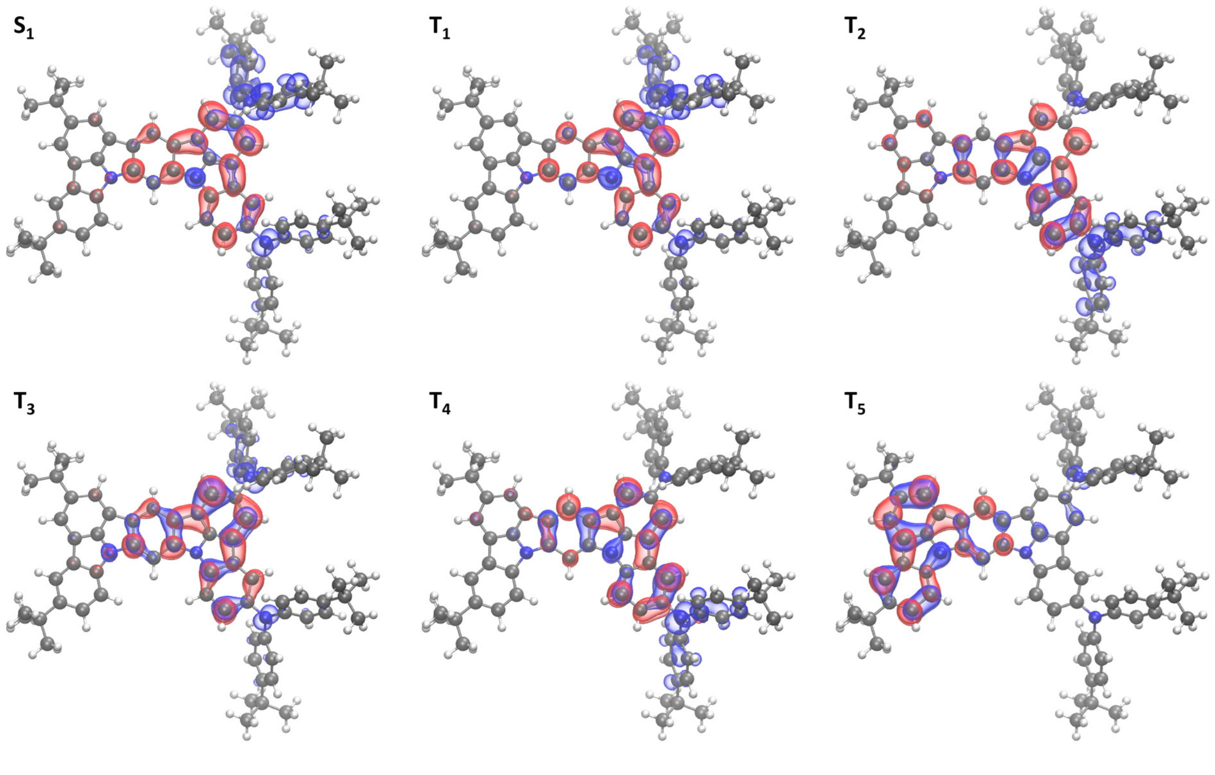

Supplementary Fig. 3 Natural transition orbital distributions of 2,5-tDPAtDIDCz. The visualized natural transition orbital distributions were calculated at optimized ground state geometry, including 1st singlet excited state (S_1) and from 1st triplet excited state (T_1) to 5th triplet excited state (T_5). Blue region represents hole occupied region and red region represents electron occupied region.

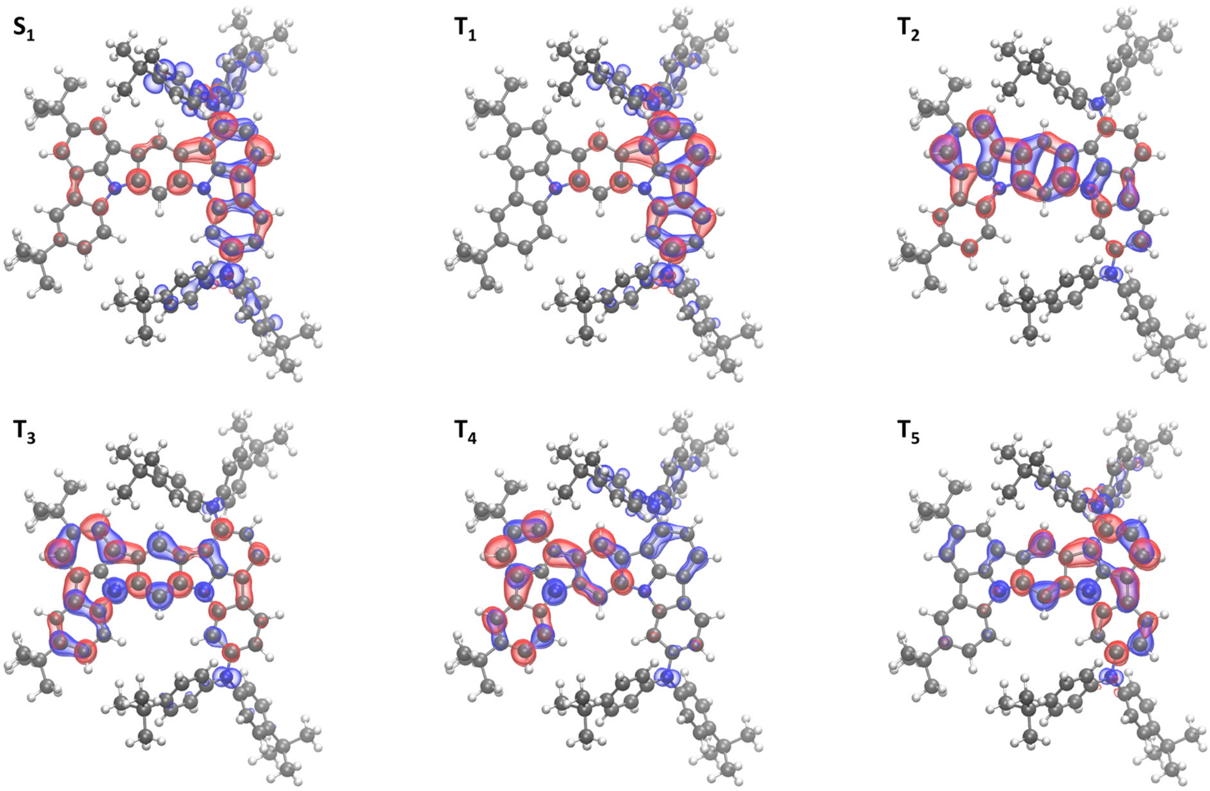

Supplementary Fig. 4 Natural transition orbital distributions of 1,6-tDPAtDIDCz. The visualized natural transition orbital distributions were calculated at optimized ground state geometry, including 1st singlet excited state (S_1) and from 1st triplet excited state (T_1) to 5th triplet excited state (T_5). Blue region represents hole occupied region and red region represents electron occupied region.

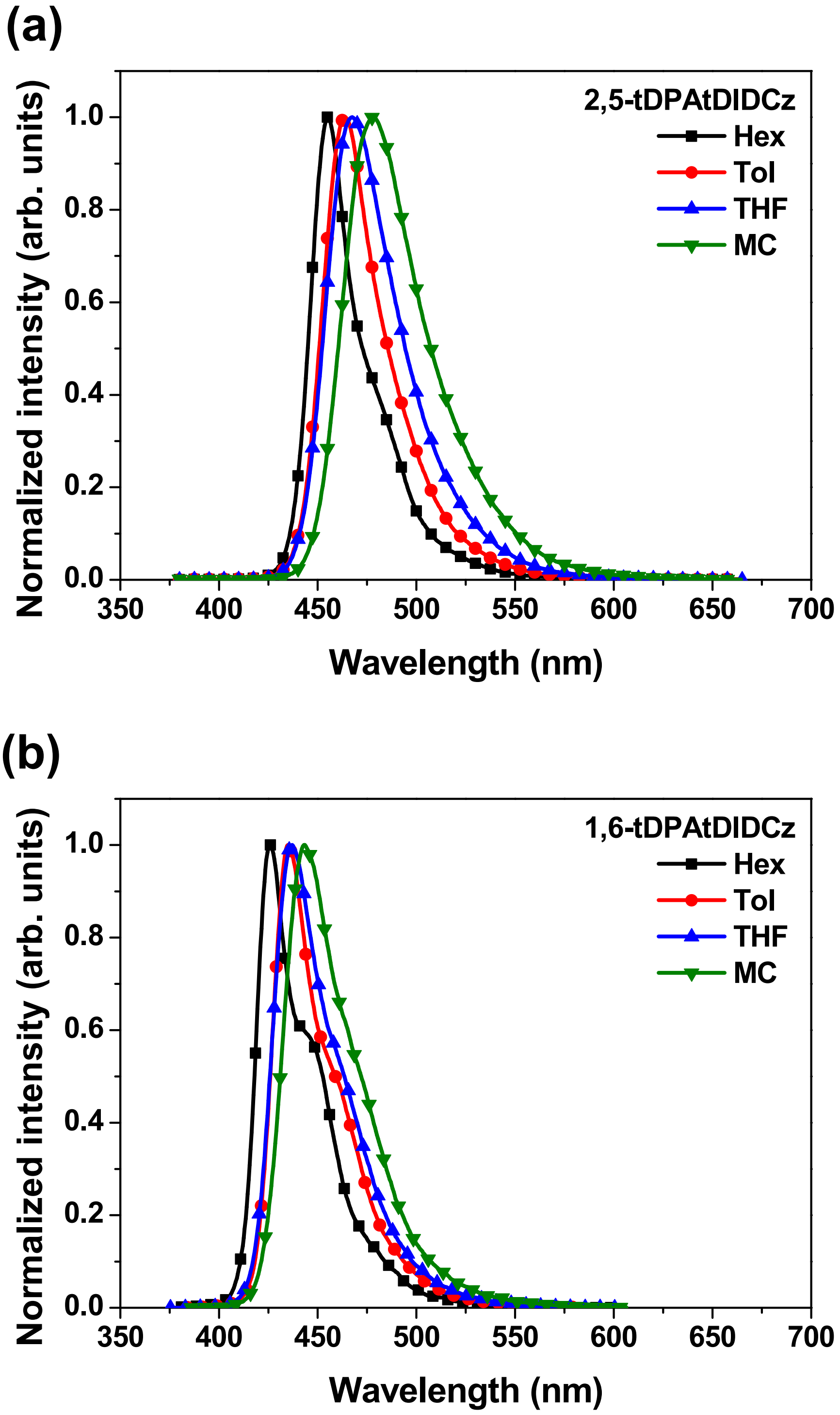

Supplementary Fig. 5 Solvatochromism. Solvent-dependent photoluminescence spectra of (a) 2,5-tDPAtDIDCz and (b) 1,6-tDPAtDIDCz measured in *n*-hexane (Hex), toluene (Tol), tetrahydrofuran (THF) and methylene chloride (MC) solution.

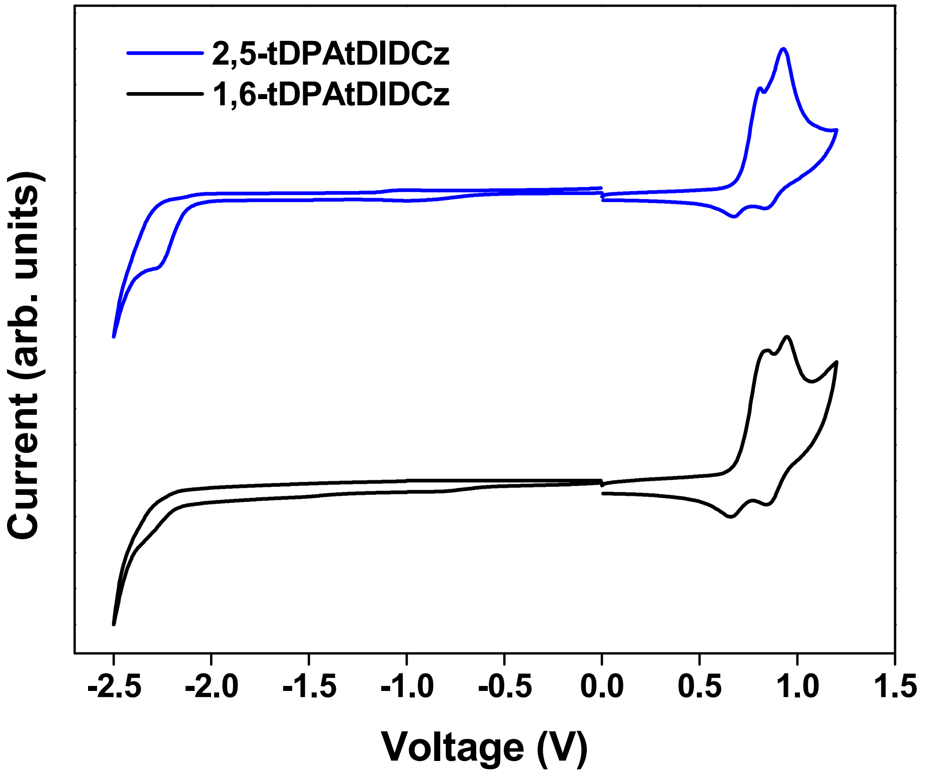

Supplementary Fig. 6 Cyclic voltammogram. The measured oxidation and reduction scan of 2,5-tDPAtDIDCz (blue) and 1,6-tDPAtDIDCz (black).

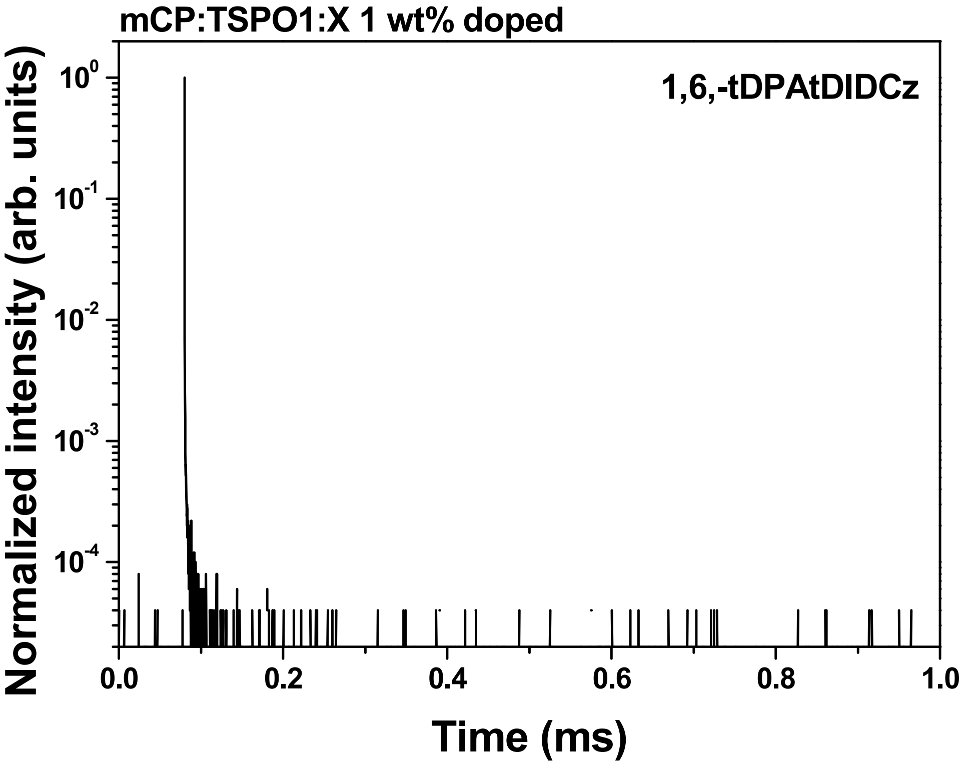

Supplementary Fig. 7 Transient photoluminescence decay curve. Detected decay curve during 1 ms time interval of 1,6-tDPAtDIDCz doped in mCP:TSPO1 host.

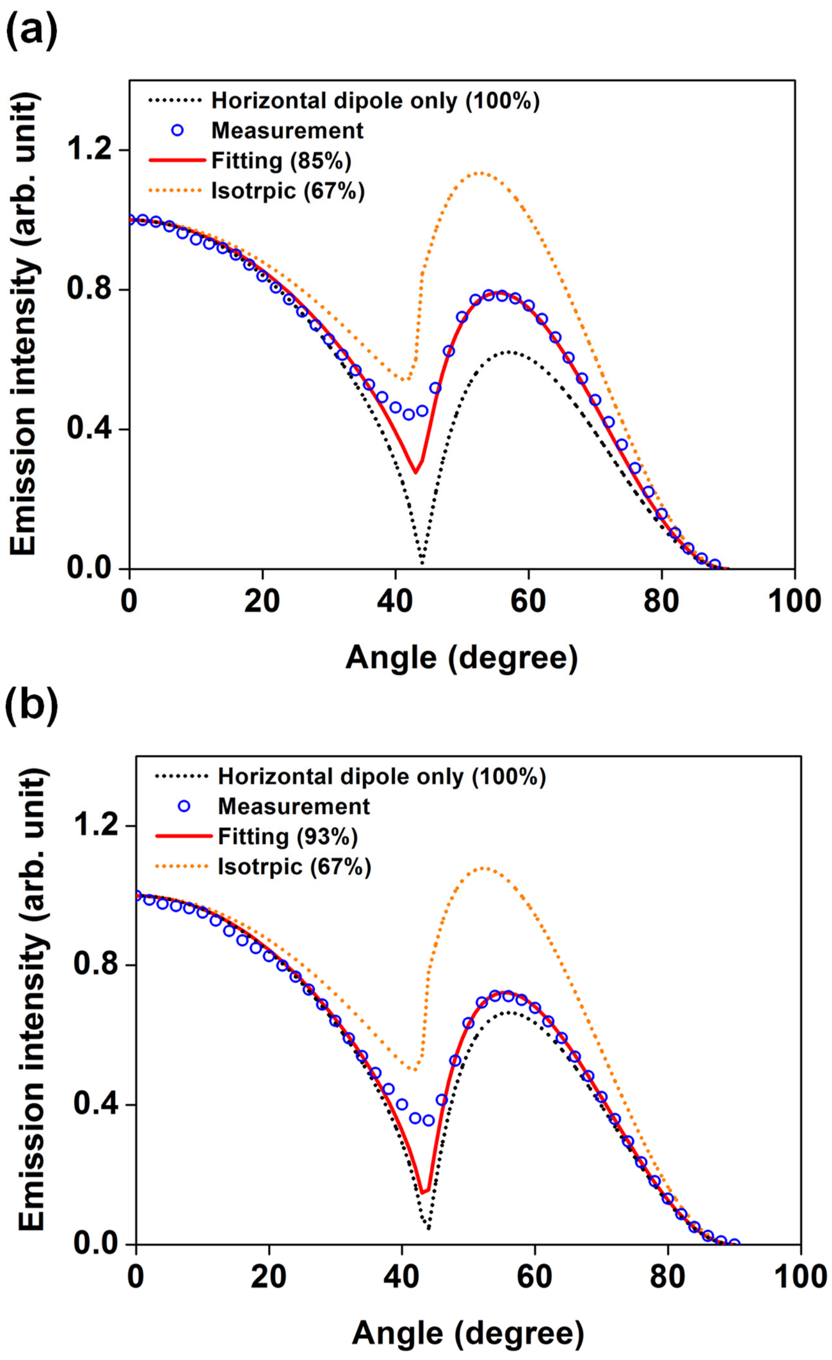

Supplementary Fig. 8 Angle-dependent photoluminescence spectra. Measured angle-dependent photoluminescence spectra and fitted horizontal emitting dipole orientation ratio of 2,5-tDPAtDIDCz doped in (a) mCP:TSPO1 matrix and (b) mBisPCz-O-BN matrix.

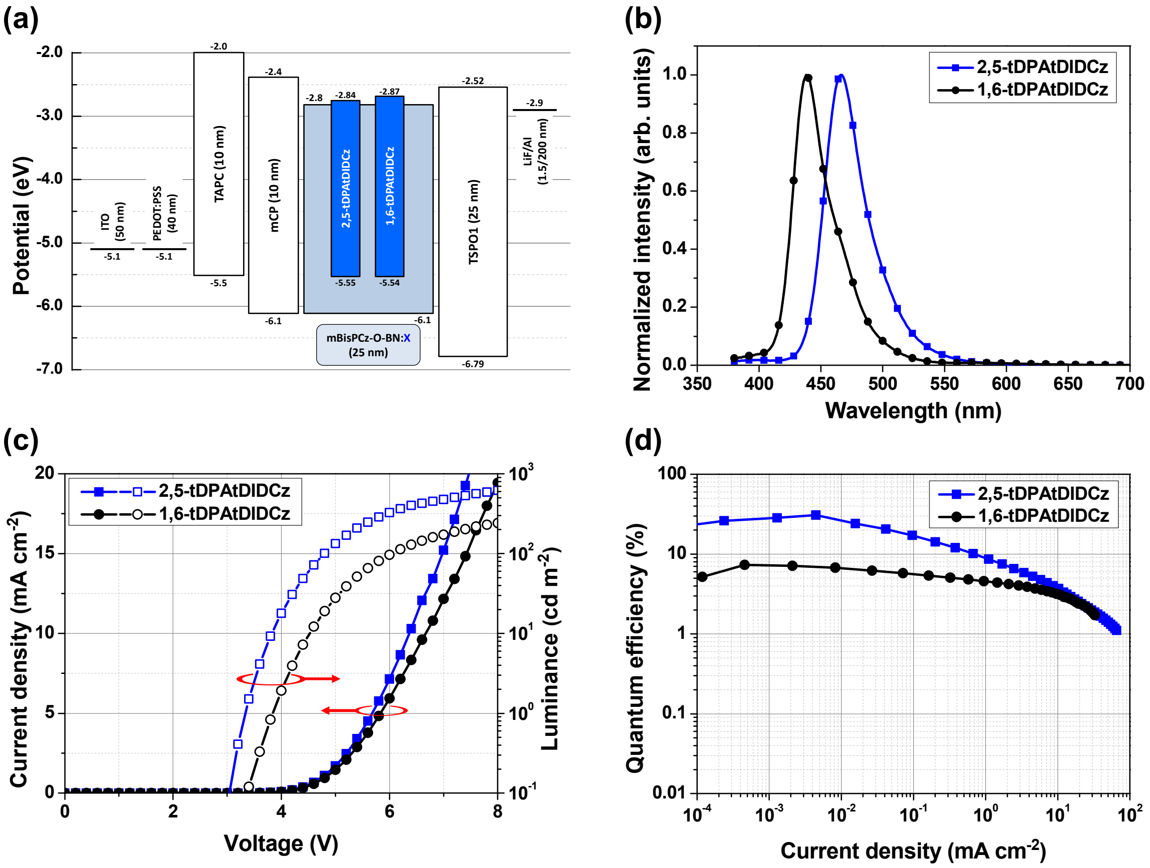

Supplementary Fig. 9 OLED device performance. (a) The device structure and energy level diagram of mBisPCz-O-BN hosted blue OLEDs. (b) Normalized electroluminescence spectra. (c) Current density–voltage–luminance curves. (d) External quantum efficiency–current density curves of 2,5-tDPAtDIDCz (blue) and 1,6-tDPAtDIDCz (black) devices.

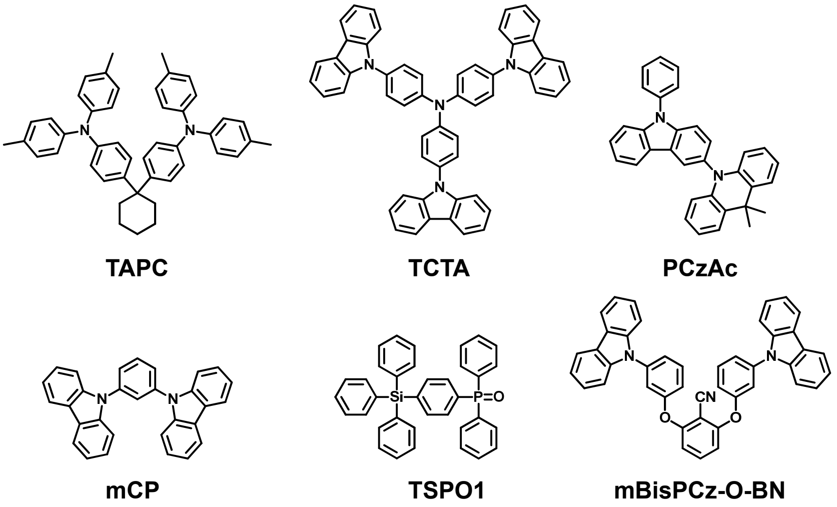

Supplementary Fig. 10 Chemical structures. The structure of materials used in blue OLED devices.

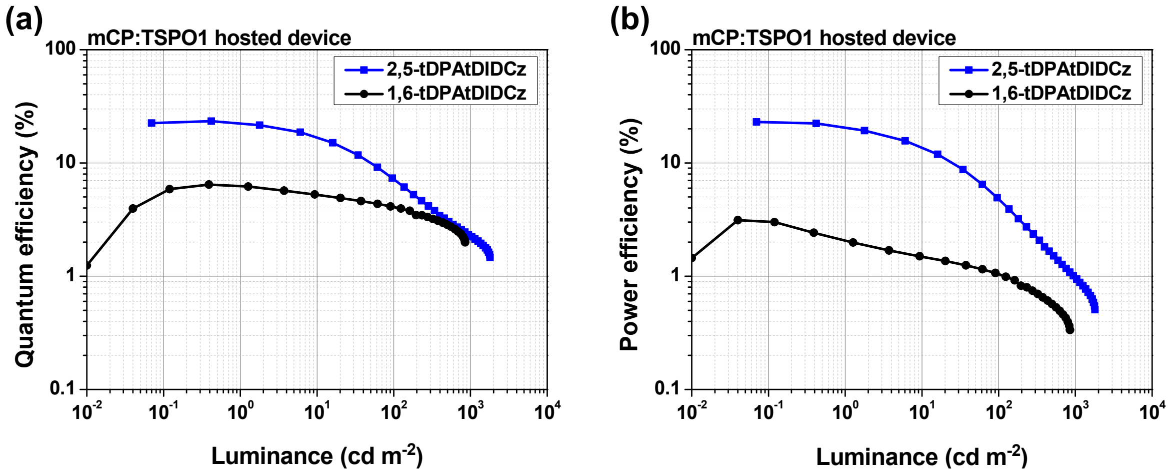

Supplementary Fig. 11 OLED device performance. The device performance of mCP:TSPO1 hosted devices. (a) External quantum efficiency–luminance curves and (b) power efficiency–luminance curves of 2,5-tDPAtDIDCz (blue) and 1,6-tDPAtDIDCz (black).

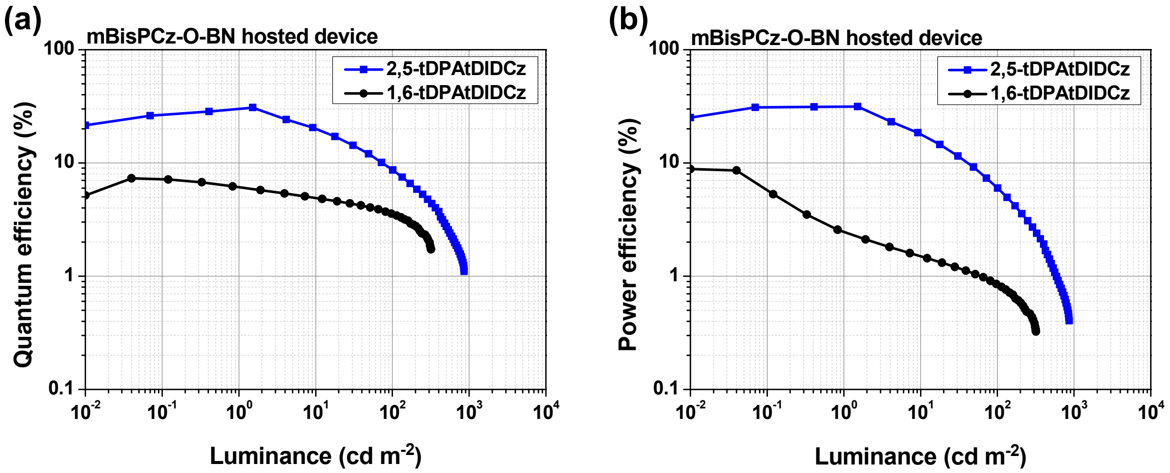

Supplementary Fig. 12 OLED device performance. The device performance of mBisPCz-O-BN hosted devices. (a) External quantum efficiency–luminance curves and (b) power efficiency–luminance curves of 2,5-tDPAtDIDCz (blue) and 1,6-tDPAtDIDCz (black).

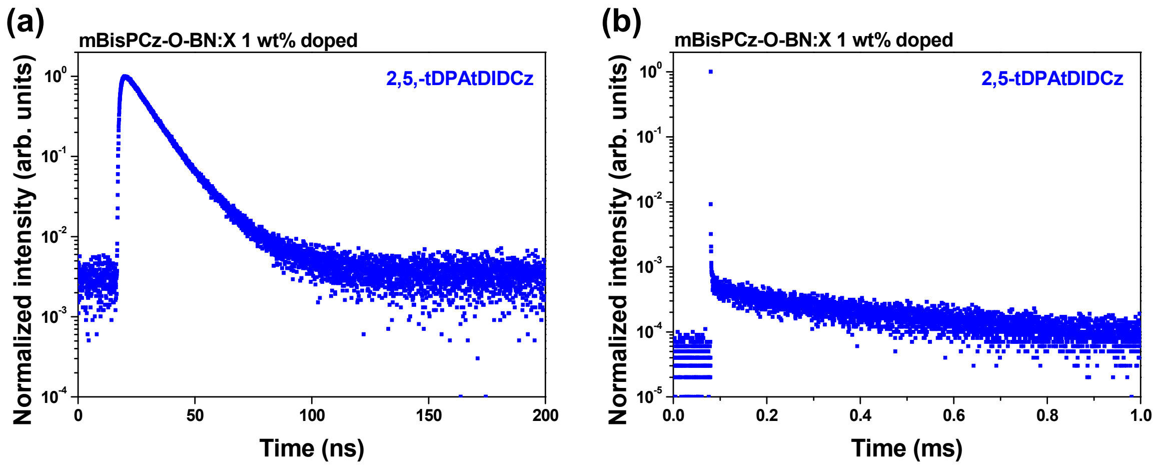

Supplementary Fig. 13 Transient photoluminescence decay curve. (a) Prompt component decay and (b) delayed component decay curves of 2,5-tDPAtDIDCz doped in mBisPCz-O-BN host.

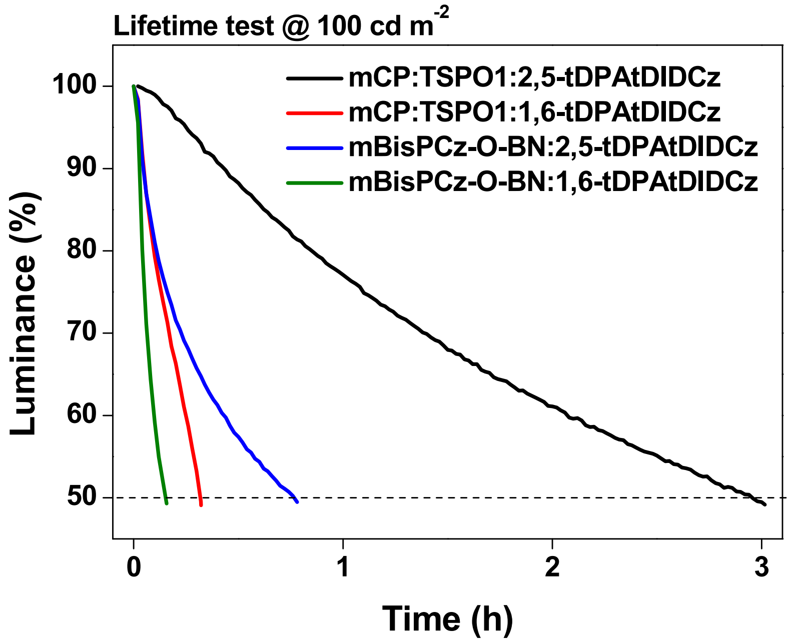

Supplementary Fig. 14 Device lifetime. Luminance–lifetime curves of four MR-TADF OLEDs.

Supplementary Table 1 The ground state three dimensional coodinates of 2,5-tDPAtDIDCz and 1,6-tDPAtDIDCz optimized at B3LYP/6-31G(*d*,*p*) level.

	Optimized S₀ Geometry						
	**2,5-tDPAtDIDCz**				**1,6-tDPAtDIDCz**		
	x	*y*	*z*		*x*	*y*	*z*
C	-10.769210	4.812393	-1.363154	C	2.364117	8.801316	-1.472356
C	-9.320430	5.319343	-1.233558	C	2.762586	8.028120	-0.192753
C	-8.983220	6.135565	-2.504068	C	4.288896	7.821554	-0.216367
C	-9.245876	6.245035	0.004031	C	2.414621	8.882312	1.049538
C	-8.308885	4.166977	-1.077316	C	1.989384	6.695551	-0.135153
C	-6.936143	4.464489	-0.946379	C	2.636475	5.447311	-0.110708
C	-5.957702	3.481860	-0.800750	C	1.940148	4.234913	-0.060460
C	-8.697198	2.820616	-1.059339	C	0.553825	4.276149	-0.033037
C	-7.750915	1.804059	-0.915683	C	-0.149504	5.531600	-0.054013
C	-6.364498	2.152741	-0.785255	C	-1.590511	5.234723	-0.012402
N	-5.628176	0.961909	-0.656144	C	-1.628468	3.853363	0.029872
C	-6.533170	-0.062228	-0.706764	C	-2.699764	2.983582	0.076068
C	-7.847836	0.337061	-0.861998	C	-2.064138	1.658266	0.094946
C	-8.760311	-0.723611	-0.910714	C	-2.576148	0.361370	0.135047
C	-8.300816	-2.064842	-0.803243	C	-1.689728	-0.718582	0.135337
C	-9.357421	-3.193059	-0.863527	C	-1.873755	-2.177631	0.148724
C	-10.371097	-3.017992	0.292678	C	-0.577558	-2.672103	0.123117
C	-10.106983	-3.131173	-2.216056	N	0.387578	-1.701636	0.097051
C	-8.736762	-4.597621	-0.734709	C	-0.076048	-3.962551	0.038753
C	-6.923710	-2.363914	-0.645739	C	1.373652	-3.768421	-0.036828
C	-5.983357	-1.323260	-0.593174	C	1.626825	-2.352723	-0.007617
C	-4.545229	-1.057061	-0.452984	C	2.908025	-1.823410	-0.055127
C	-4.363657	0.379893	-0.496317	C	3.992107	-2.711651	-0.167341
C	-3.115968	0.988840	-0.389908	N	5.314482	-2.203498	-0.239450
C	-2.030539	0.130281	-0.237107	C	5.581078	-1.015529	-0.975351
N	-0.667820	0.431667	-0.103714	C	6.413765	-0.014838	-0.450400
C	-0.005759	-0.758164	0.017649	C	6.669686	1.142459	-1.177678
C	1.366219	-0.642043	0.158304	C	6.102811	1.370983	-2.443707
C	2.033998	-1.865495	0.297800	C	6.408589	2.673166	-3.204943
C	1.276309	-3.063129	0.252574	C	5.937558	3.885560	-2.366167
N	1.967038	-4.309643	0.377083	C	7.932693	2.783154	-3.447942
C	1.539679	-5.249529	1.349390	C	5.699494	2.731424	-4.570970
C	1.549105	-6.626270	1.086032	C	5.268568	0.364624	-2.949490
C	1.123351	-7.539520	2.050161	C	5.016966	-0.809777	-2.240483
C	0.652674	-7.130182	3.305033	C	6.372540	-2.856980	0.449881
C	0.167257	-8.111693	4.386279	C	7.630247	-3.010800	-0.145912

C	0.257088	-9.578663	3.925477	C	8.670132	-3.638911	0.539415
C	1.035912	-7.952271	5.656733	C	8.500721	-4.155123	1.831106
C	-1.308630	-7.807129	4.736822	C	9.623277	-4.863114	2.610198
C	0.639551	-5.746077	3.548683	C	10.936244	-4.932712	1.808007
C	1.076850	-4.823160	2.605474	C	9.902351	-4.096761	3.925064
C	3.069762	-4.582424	-0.472559	C	9.186371	-6.309081	2.945311
C	3.059023	-4.171913	-1.816003	C	7.229215	-3.999150	2.410235
C	4.149632	-4.422571	-2.640760	C	6.187939	-3.359187	1.747835
C	5.292834	-5.101729	-2.185560	C	3.760974	-4.102742	-0.209779
C	6.470501	-5.362550	-3.141138	C	2.472233	-4.624763	-0.133967
C	5.986418	-6.216749	-4.336963	C	-1.075975	-4.947643	0.019401
C	7.018733	-4.014547	-3.666767	C	-2.419128	-4.542716	0.081627
C	7.625708	-6.113305	-2.452424	C	-2.859815	-3.191624	0.133576
C	5.284955	-5.510657	-0.845264	N	-4.251969	-2.912415	0.156166
C	4.206557	-5.252079	0.000403	C	-4.793360	-2.058803	1.150266
C	-0.134013	-3.088895	0.088549	C	-5.924538	-1.267654	0.880282
C	-0.823137	-1.875620	-0.020274	C	-6.444714	-0.423827	1.854974
C	1.588834	0.810541	0.122828	C	-5.866066	-0.305786	3.131285
C	0.306121	1.442994	-0.038965	C	-6.473944	0.650027	4.172511
C	0.190236	2.827246	-0.103699	C	-6.485960	2.091186	3.608915
C	1.352092	3.592645	-0.018216	C	-7.924806	0.214140	4.487171
C	2.618349	2.999080	0.155041	C	-5.677504	0.658657	5.490813
N	3.779330	3.816897	0.249053	C	-4.736164	-1.094789	3.379723
C	3.766685	4.969337	1.078555	C	-4.211521	-1.961129	2.420240
C	3.128531	4.955475	2.325476	C	-5.113228	-3.624300	-0.726638
C	3.106922	6.096444	3.127566	C	-4.780231	-3.765128	-2.078924
C	3.730435	7.289118	2.737694	C	-5.611741	-4.472837	-2.946087
C	3.734104	8.561577	3.603134	C	-6.811894	-5.052608	-2.512189
C	3.050301	9.714331	2.830078	C	-7.757449	-5.830295	-3.444794
C	2.983007	8.364810	4.933376	C	-7.232677	-5.888624	-4.891718
C	5.192957	8.961053	3.928824	C	-7.912390	-7.281120	-2.929799
C	4.372932	7.282951	1.487230	C	-9.144242	-5.144470	-3.462961
C	4.387939	6.160218	0.667759	C	-7.132991	-4.895802	-1.152797
C	4.951515	3.464367	-0.472143	C	-6.305476	-4.207988	-0.271676
C	6.222684	3.586349	0.112021	C	-0.265732	-0.463827	0.095306
C	7.363763	3.241596	-0.603370	C	0.280929	0.815597	0.058141
C	7.302393	2.744272	-1.916746	C	-0.632232	1.865573	0.057043
C	8.594604	2.365165	-2.661509	N	-0.402351	3.247454	0.018894
C	9.512104	3.606258	-2.769743	C	-3.951589	3.618513	0.082332
C	9.332605	1.251708	-1.880680	C	-4.026301	5.034252	0.040461
C	8.317231	1.848688	-4.085826	C	-5.392825	5.759314	0.042064

C	6.025717	2.619231	-2.480698	C	-5.493257	6.675579	1.285107
C	4.872431	2.978109	-1.783317	C	-5.528579	6.616959	-1.239018
C	2.729623	1.602137	0.231262	C	-6.584496	4.783171	0.080193
C	-2.168187	-1.310167	-0.189652	C	-2.853994	5.837440	-0.005880
C	-3.434044	-1.885606	-0.299234	C	0.583930	6.713512	-0.105251
H	-11.448798	5.663713	-1.471392	H	2.900159	9.755785	-1.525451
H	-11.085099	4.249547	-0.478688	H	1.292655	9.020452	-1.498858
H	-10.900480	4.172735	-2.242056	H	2.607971	8.223828	-2.369989
H	-9.691930	6.962467	-2.626321	H	4.791092	8.793385	-0.259341
H	-9.037009	5.506576	-3.398564	H	4.609582	7.246069	-1.090895
H	-7.977876	6.564454	-2.458662	H	4.644860	7.306710	0.682003
H	-9.957787	7.072393	-0.094216	H	2.952862	9.836567	1.020722
H	-8.249336	6.678328	0.130115	H	2.693125	8.362429	1.971931
H	-9.488153	5.694799	0.919008	H	1.345186	9.106157	1.103807
H	-6.614294	5.501066	-0.957980	H	3.718564	5.401849	-0.129470
H	-4.912545	3.755764	-0.703200	H	2.480347	3.294272	-0.042119
H	-9.741447	2.547940	-1.157115	H	-3.646677	0.195437	0.169651
H	-9.822271	-0.535449	-1.030027	H	3.082770	-0.755715	-0.009990
H	-10.894036	-2.058912	0.237260	H	6.859501	-0.150673	0.529438
H	-9.867556	-3.066578	1.263661	H	7.319672	1.891344	-0.734327
H	-11.127339	-3.810677	0.261582	H	6.433812	3.922111	-1.391619
H	-10.621150	-2.175957	-2.356039	H	4.857087	3.846464	-2.192385
H	-10.860459	-3.925101	-2.272500	H	6.162748	4.821932	-2.889426
H	-9.413110	-3.261057	-3.052951	H	8.495140	2.790913	-2.509635
H	-8.216026	-4.729059	0.219375	H	8.168535	3.708780	-3.985177
H	-8.030406	-4.808840	-1.544132	H	8.294730	1.941059	-4.046686
H	-9.527593	-5.352992	-0.783805	H	4.610463	2.687924	-4.467361
H	-6.604708	-3.395500	-0.566351	H	6.014035	1.914165	-5.228191
H	-2.997416	2.064833	-0.424209	H	5.944962	3.672030	-5.074404
H	3.107772	-1.934697	0.433769	H	4.807248	0.477026	-3.923884
H	1.892989	-6.984157	0.121453	H	4.375308	-1.572428	-2.669354
H	1.151572	-8.592926	1.795404	H	7.794943	-2.634884	-1.150309
H	1.285924	-9.869014	3.688731	H	9.625225	-3.732760	0.035380
H	-0.362922	-9.767010	3.042854	H	11.316943	-3.935956	1.562240
H	-0.096540	-10.239177	4.723826	H	10.814279	-5.492155	0.874748
H	2.086616	-8.172792	5.441880	H	11.703798	-5.442065	2.399544
H	0.697506	-8.638800	6.441286	H	10.223451	-3.070518	3.718971
H	0.984536	-6.936323	6.059273	H	10.695170	-4.593067	4.496207
H	-1.950764	-7.921326	3.857357	H	9.015151	-4.046500	4.563132
H	-1.435209	-6.787019	5.111186	H	8.990631	-6.880057	2.031835
H	-1.668154	-8.493443	5.512114	H	8.276120	-6.328629	3.551830

H	0.291003	-5.369949	4.506357	H	9.972410	-6.825843	3.507646
H	1.061597	-3.763595	2.837572	H	7.043529	-4.372927	3.413023
H	2.190629	-3.654136	-2.209239	H	5.224188	-3.246029	2.233024
H	4.096151	-4.087211	-3.672644	H	4.608759	-4.771270	-0.306134
H	5.601726	-7.183512	-3.996179	H	2.326228	-5.700344	-0.170111
H	6.811984	-6.405050	-5.032928	H	-0.845017	-6.007338	-0.031984
H	5.188065	-5.718904	-4.895183	H	-3.187189	-5.308693	0.083410
H	6.253743	-3.447016	-4.204842	H	-6.393408	-1.319136	-0.096561
H	7.854665	-4.182909	-4.355439	H	-7.321444	0.166031	1.603010
H	7.377939	-3.390538	-2.841892	H	-7.087758	2.164651	2.697870
H	7.310538	-7.094180	-2.081737	H	-5.471972	2.426943	3.368715
H	8.035877	-5.545509	-1.610854	H	-6.910871	2.785387	4.342845
H	8.438605	-6.276722	-3.167405	H	-8.554021	0.222569	3.592083
H	6.139637	-6.031840	-0.429096	H	-8.378408	0.891200	5.220057
H	4.246301	-5.573054	1.035857	H	-7.947500	-0.799283	4.901013
H	-0.633412	-4.051253	0.057432	H	-4.642971	0.983125	5.338904
H	-0.772968	3.308781	-0.233339	H	-5.660823	-0.328921	5.963302
H	1.286170	4.673124	-0.081989	H	-6.141514	1.353797	6.197944
H	2.646064	4.046470	2.669051	H	-4.244514	-1.054363	4.344868
H	2.599677	6.033319	4.083529	H	-3.342348	-2.563959	2.659220
H	2.010730	9.464283	2.594341	H	-3.863733	-3.318711	-2.450615
H	3.562275	9.932400	1.888175	H	-5.310418	-4.554324	-3.984074
H	3.051932	10.631609	3.429899	H	-6.262342	-6.392025	-4.953458
H	1.930706	8.108169	4.773183	H	-7.128276	-4.889753	-5.327800
H	3.011288	9.292405	5.514173	H	-7.935182	-6.449286	-5.516756
H	3.437384	7.578767	5.545336	H	-6.946318	-7.796088	-2.915220
H	5.773148	9.156450	3.022175	H	-8.589893	-7.847197	-3.579153
H	5.701016	8.166422	4.484859	H	-8.321569	-7.311583	-1.915659
H	5.213691	9.870690	4.539839	H	-9.067408	-4.116556	-3.832102
H	4.865803	8.182423	1.129469	H	-9.592887	-5.107791	-2.465885
H	4.882846	6.199505	-0.296874	H	-9.832613	-5.690574	-4.117972
H	6.310599	3.953104	1.129252	H	-8.046487	-5.333007	-0.760132
H	8.326424	3.351121	-0.112284	H	-6.580467	-4.119358	0.774070
H	9.781161	4.000285	-1.785283	H	1.350307	0.982909	0.025600
H	9.019199	4.409580	-3.326994	H	-4.861165	3.032366	0.118387
H	10.441545	3.350992	-3.291425	H	-4.703743	7.432478	1.300057
H	9.595560	1.570988	-0.867864	H	-5.411293	6.091397	2.207465
H	10.260616	0.974863	-2.393871	H	-6.456061	7.199284	1.298384
H	8.710077	0.355021	-1.796493	H	-4.743540	7.375249	-1.309968
H	7.818929	2.603489	-4.702984	H	-6.493641	7.136441	-1.251033
H	7.695394	0.947574	-4.078684	H	-5.468229	5.991148	-2.135364

H	9.262011	1.593158	-4.576423	H	-6.577022	4.164596	0.983436
H	5.910281	2.247078	-3.492249	H	-6.597569	4.119970	-0.790845
H	3.902316	2.878292	-2.258804	H	-7.521593	5.349289	0.076863
H	3.705095	1.148139	0.368779	H	-2.952488	6.917653	-0.037103
H	-3.552281	-2.964476	-0.264167	H	0.051473	7.659781	-0.121685

Supplementary Table 2 The vertical excitation energy gap and spin-orbit coupling matrix element of two emitters between different excited states.

n	2,5-tDPAtDIDCz				1,6-tDPAtDIDCz			
	Btw 1st singlet state		Btw 1st triplet state		Btw 1st singlet state		Btw 1st triplet state	
	E_{S1} - E_{Tn} (eV)^{a}	ξ(T_n-S_1) (cm^{-1})^{b}	E_{Tn} - E_{T1} (eV)^{c}	ξ(T_n-T_1) (cm^{-1})^{d}	E_{S1} - E_{Tn} (eV)$^{a\ a}$	ξ(T_n-S_1) (cm^{-1})^{b}	E_{Tn} - E_{T1} (eV)^{c}	ξ(T_n-T_1) (cm^{-1})^{d}
1	0.294	0.378	0	-	0.545	0.512	0	-
2	0.110	0.169	0.184	0.251	0.158	0.121	0.387	0.502
3	0.006	0.835	0.288	0.229	-0.01	0.254	0.555	0.432
4	-0.108	0.755	0.402	0.371	-0.077	0.200	0.622	0.163
5	-0.225	0.271	0.519	0.441	-0.124	0.665	0.669	1.128

[a] Veritcal excitation energy gap between 1st singlet state and corresponding nth triplet state. [b] Spin-orbit coupling matrix elements between 1st singlet state and corresponding nth triplet states. [c] Veritcal excitation energy gap between 1st triplet state and corresponding nth triplet state. [d] Spin-orbit coupling matrix elements between 1st triplet state and corresponding nth triplet states.

Supplementary Table 3 Summary of photophysical properties related to radiative transition in solid matrix.

	$λ_{em}$ (nm)	E_S (eV)	FWHM (nm)	PLQY (%)	$τ_{PF}$ (ns)	k_{PF} (10^7 s^{-1})	$τ_{DF}$ (μs)	k_{DF} (10^3 s^{-1})	$Φ_{PF}$ (%)	$Φ_{DF}$ (%)	k_{ISC} (10^7 s^{-1})	k_{RISC} (10^4 s^{-1})
**2,5-tDPAtDIDCz**[a]	461	2.69	40	88	9.0	11.11	251.4	3.98	26	62	8.22	1.28
**2,5-tDPAtDIDCz**[b]	464	2.67	42	92	11.6	8.62	240.5	4.16	29	63	6.12	1.27
**1,6-tDPAtDIDCz**[a]	437	2.84	40	92	3.0	33.33	N.A.[c]	N.A.[c]	92	N.A.[c]	N.A.[c]	N.A.[c]

[a] Measured at mCP:TSPO1 blended film with 1 wt% doping concentration under N_2 atmosphere. [b] Measured at mBisPCz-O-BN blended film with 1 wt% doping concentration under N_2 atmosphere. [c] N.A.: Not assigned

Supplementary Table 4 Summarized device performances of reported blue OLEDs.

Emitter	Emitter type	λ_{EL}[a] (nm)	$FWHM_{EL}$[b] (nm)	EQE_{Max}[c] (%)	CIE (x, y)[d]	Ref
**CzBPCN**	**DA-TADF**[e]	460	48	14	(0.14, 0.12)	1
**TDBA-Ac**		445	48	21.5	(0.15, 0.06)	2
		464	55	25.7	(0.14, 0.15)	
**TDBA-Ac**		465	56	32.2	(0.14, .0.15)	
**DMAC2PTO**		448	52	15.2	(0.154, 0.108)	3
**TDBA-SAF**		456	55	28.2	(0.142, 0.090)	4
**TDBA-PAS**		435	50	22.35	(0.155, 0.042)	5
**TDBA-DPAC**		449	59	21.32	(0.150, 0.077)	
**DABNA-1**	**MR-TADF**[f]	459	28	13.5	(0.13, 0.09)	6
**DABNA-TP-TB**		457	33	19.5	(0.14, 0.11)	7
**ν-DABNA**		469	18	34.4	(0.12, 0.11)	8
**V-DABNA-F8**		468	15	26.6	(0.09, 0.10)	9
**ν-DABNA-O-Me**		465	23	29.5	(0.13, 0.10)	10
**2B-DTACrs**		447	26	14.8	(0.150, 0.044)	11
**m-ν-DABNA**		471	18	36.2	(0.12, 0.12)	12
**4F-ν-DABNA**		464	18	35.8	(0.13, 0.10)	
**4F-m-ν-DABNA**		461	18	33.7	(0.13, 0.06)	
**BOBO-Z**		445	18	13.6	(0.15, 0.04)	13
**BOBS-Z**		456	23	26.9	(0.14, 0.06)	
**BSBS-Z**		463	22	26.8	(0.13, 0.08)	
**t-DAB-DPA**		459	26	27.9	(0.13, 0.08)	14
**3tPAB**		460	26	19.3	(0.14, 0.08)	15
**m-DINBO**		466	21	24.2	(0.126, 0.098)	16
**BBCz-DB**		469	27	29.3	(0.12, 0.18)	17
**QA-1**		455	39	17.1	(0.14, 0.12)	18
**CzBO**		448	30	13.4	(0.15, 0.05)	19
**CzBS**		473	31	23.1	(0.11, 0.16)	

**tCBNDADPO**		468	24	13.8	(0.12, 0.16)	20
**mBP-DABNA-Me**		468	28	24.3	(0.12, 0.14)	21
**R-DOBN**		464	35	25.6	(0.13, 0.12)	22
**tDIDCz**		401	14	2.75	(0.164, 0.018)	23
**BisICz**		437	24	6.5	(0.16, 0.04)	
**tBisICz**		445	22	15.1	(0.16, 0.05)	24
**tPBisICz**		452	21	23.1	(0.15, 0.05)	
**t3IDCz**	**ICz**	472	25	30.0	(0.119, 0.161)	25
**p3IDCz**	**derivatives**[g]	472	23	30.9	(0.120, 0.158)	
**2,5-tDPAtDIDCz**		464	36	23.4	(0.13, 0.12)	
		466	38	30.8	(0.13, 0.14)	this
**1,6-tDPAtDIDCz**		437	32	6.4	(0.16, 0.04)	work
		438	36	7.3	(0.16, 0.05)	

[a] Peak wavelength of electroluminescence (EL). [b] Full-width-at-half-maximum of EL spectrum. [c] Maximum external quantum efficiency. [d] CIE color coordinates. [e] Donor-acceptor type TADF. [f] Multiple-resonance type TADF. [g] Indolo[3,2,1-*jk*]carbazole derived MR type emitters.

Supplementary Note | Synthesis of Two Emitters.

Synthesis of *tert*-butyl 3,6-dibromo-9*H*-carbazole-9-carboxylate

Synthetic procedure of *tert*-butyl 3,6-dibromo-9*H*-carbazole-9-carboxylate was the same as that reported in the literature.[26]

Synthesis of N^3,N^3,N^6,N^6-tetrakis(4-(*tert*-butyl)phenyl)-9*H*-carbazole-3,6-diamine

Tert-butyl 3,6-dibromo-9*H*-carbazole-9-carboxylate (10.00 g, 23.52 mmol), bis(4-(*tert*-butyl)phenyl)amine (19.86 g, 70.57 mmol), sodium *tert*-butoxide (NaOtBu) (13.56 g, 141.14 mmol), tris(dibenzylideneacetone)dipalladium ($Pd_2(dba)_3$) (2.15 g, 2.35 mmol), 2-dicyclohexylphosphino-2′,4′,6′-triisopropylbiphenyl (XPhos) (2.24 g, 4.70 mmol) and toluene (200 mL) were added into a 500 mL round-bottom (RB) flask. The mixture was stirred and refluxed under a nitrogen purged condition for 6 h. The reaction was monitored using thin layer chromatography (TLC). After full consumption of starting material, additional addition of sodium *tert*-butoxide (NaOtBu) (13.56 g, 141.14 mmol) was proceeded to deprotect *tert*-butyloxycarbonyl (BOC). The reaction was kept for overnight and allowed to cooled down to room temperature. Excess methylene chloride (MC) was added, and the reaction solution was filtered by silica gel/celite packed filter. The filtrate solution was completely concentrated by rotary evaporator and adsorbed into silica gel for column purification. With eluent of MC and *n*-hexane (2:1), the column purification using silica gel was proceeded and re-precipitated by MC and *n*-hexane. After filtration, a white solid was obtained. (10.7 g, Yield 63%)

^{1}H NMR (300 MHz, DMSO-d_6) : δ 11.28 (s, 1H), 7.82 (s, 2H), 7.46 (d, J = 8.1 Hz, 2H), 7.20 (d, J = 7.2 Hz, 8H), 7.12 (d, J = 7.5 Hz, 2H), 6.84 (d, J = 7.2 Hz, 8H), 1.22 (s, 36H)

MS (APCI) m/z : Found 726.38 [$(M + H)^+$]. Calculated For $C_{52}H_{59}N_3$: 725.47

Synthesis of *tert*-butyl 2,7-dibromo-9*H*-carbazole-9-carboxylate

The synthetic method of *tert*-butyl 2,7-dibromo-9*H*-carbazole-9-carboxylate was exactly the same as that of *tert*-butyl 2,7-dibromo-9*H*-carbazole-9-carboxylate except for starting material. 2,7-Dibromo-9*H*-carbazole (6.00 g, 18.46 mmol) was used instead of 3,6-dibromo-9*H*-carbazole. After purification, a white solid was obtained. (7.50 g, Yield 96%)

^{1}H NMR (300 MHz, $CDCl_3$) : δ 8.49 (s, 2H), 7.77 (d, *J* = 8.1 Hz, 2H), 7.47 (dd, *J* = 8.2, 1.7 Hz, 2H), 1.77 (s, 9H)

MS (APCI) m/z : Found 424.53 [(M + H)$^+$]. Calculated For $C_{17}H_{15}Br_2NO_2$: 422.95

Synthesis of N^2,N^2,N^7,N^7-tetrakis(4-(*tert*-butyl)phenyl)-9*H*-carbazole-2,7-diamine

N^2,N^2,N^7,N^7-tetrakis(4-(*tert*-butyl)phenyl)-9*H*-carbazole-2,7-diamine was prepared by following the synthetic method of N^3,N^3,N^6,N^6-tetrakis(4-(*tert*-butyl)phenyl)-9*H*-carbazole-3,6-diamine. As a starting material, *tert*-butyl 2,7-dibromo-9*H*-carbazole-9-carboxylate (2.00 g, 4.70 mmol) was used. After wet purification process, a white solid was collected. (2.61 g, Yield 76%)

^{1}H NMR (300 MHz, DMSO-d_6) : δ 10.77 (s, 1H), 7.84 (d, *J* = 8.4 Hz, 2H), 7.29 (d, *J* = 8.4 Hz, 8H), 6.94 (d, *J* = 7.2 Hz, 10H), 6.77 (d, *J* = 8.1 Hz, 2H), 1.26 (s, 36H)

MS (APCI) m/z : Found 726.44 [(M + H)$^+$]. Calculated For $C_{52}H_{59}N_3$: 725.47

Synthesis of 3,6-di-*tert*-butyl-9-(2,4-dibromo-5-fluorophenyl)-9*H*-carbazole

Firstly, 3,6-di-*tert*-butyl-9*H*-carbazole (10.00 g, 35.79 mmol) was dissolved in *N*,*N*-dimethylformamide (DMF) (40 mL). Sodium hydride (NaH, 60-70% coated in mineral oil) (1.72 g, 71.58 mmol) was placed to 250 ml RB flask and immediately connected to a nitrogen purge line. 10 mL of DMF was added into RB flask to spread out NaH and DMF solution with 3,6-di-*tert*-butyl-9*H*-carbazole was added for activation reaction. After 30 min stirring at room temperature, 1,5-dibromo-2,4-difluorobenzene (38.92 g, 143.15 mmol) dissolved in

DMF solution (100 mL) was quickly injected into the RB flask. The reaction was stirred for 1 h by monitoring TLC, and quenched with excess of deionized water (DW) to remove residual NaH for 1 h. The extraction process was carried out with MC and DW saturated with ammonium chloride. The extracted organic layer was mixed with magnesium sulfate to remove small amount of salt and water, and it was filtered, and concentrated by rotary evaporator. The crude product was adsorbed into silica gel and purified with column chromatography using mixed solvent of MC and *n*-hexane (1:9). After that, a white powder was obtained. (13.20 g, Yield 69%)

^{1}H NMR (300 MHz, $CDCl_3$) : δ 8.14 (d, *J* = 1.8 Hz, 2H), 8.06 (d, *J* = 6.9 Hz, 1H), 7.46 (dd, *J* = 8.7, 1.8 Hz, 2H), 7.25 (d, *J* = 8.4 Hz, 1H), 7.00 (d, *J* = 8.7 Hz, 2H), 1.47 (s, 18H)

MS (APCI) m/z : Found 529.99 [$(M + H)^+$]. Calculated For $C_{26}H_{26}Br_2FN$: 529.04

Synthesis of *N*^{3},*N*^{3},*N*^{6},*N*^{6}-tetrakis(4-(*tert*-butyl)phenyl)-9-(2,4-dibromo-5-(3,6-di-*tert*-butyl-9*H*-carbazol-9-yl)phenyl)-9*H*-carbazole-3,6-diamine

NaH (60-70% coated in mineral oil) (0.19 g, 7.71 mmol) was added into 100 mL RB flask and connected to a nitrogen purge line, immediately. Small amount of DMF (2 mL) was added to disperse NaH. *N*^{3},*N*^{3},*N*^{6},*N*^{6}-Tetrakis(4-(*tert*-butyl)phenyl)-9*H*-carbazole-3,6-diamine (2.80 g, 3.86 mmol) with DMF (20 mL) was poured into RB flask and the mixture was heated at 80 °C. After complete dissolution, activation reaction was proceeded for 1 h and then 3,6-di-*tert*-butyl-9-(2,4-dibromo-5-fluorophenyl)-9*H*-carbazole (3.07 g, 5.78 mmol) was poured into the mixture. The reaction was kept for overnight and cooled down to room temperature. To quench remained NaH, excess of DW was added into solution and stirred for 30 min. Then the solution was filtered and washed with methanol several times. After drying of filtered solid, the crude product was dissolved again with small amount of MC and adsorbed into

silica gel. The adsorbed crude product was purified by column chromatography with eluent of MC: *n*-hexane (1:2). An ivory-white powder was obtained as a product. (4.40 g, Yield 92%)

^{1}H NMR (500 MHz, CD_2Cl_2) : δ 8.39 (s, 1H), 8.14 – 8.10 (m, 2H), 7.68 (s, 2H), 7.61 (s, 1H), 7.46 (dd, *J* = 8.6, 2.0 Hz, 2H), 7.17 (d, *J* = 8.6 Hz, 11H), 7.11 – 7.05 (m, 4H), 6.91 (d, *J* = 8.3 Hz, 9H) 1.42 (s, 18H), 1.24 (s, 36 H).

MS (APCI) m/z : Found 1236.01 [$(M + H)^+$]. Calculated For $C_{78}H_{84}Br_2N_4$: 1234.51

Synthesis of 13,16-di-*tert*-butyl-*N*^{2},*N*^{2},*N*^{5},*N*^{5}-tetrakis(4-(*tert*-butyl)phenyl)indolo[3,2,1-*jk*]indolo[1′,2′,3′:1,7]indolo[2,3-*b*]carbazole-2,5-diamine (2,5-tDPAtDIDCz)

N^3,*N*^{3},*N*^{6},*N*^{6}-Tetrakis(4-(*tert*-butyl)phenyl)-9-(2,4-dibromo-5-(3,6-di-*tert*-butyl-9*H*-carbazol-9-yl)phenyl)-9*H*-carbazole-3,6-diamine (2.50 g, 2.02 mmol), benzyltriethylammoium chloride ($BnEt_3NCl$) (0.92 g, 4.04 mmol), $Pd_2(dba)_3$ (0.56 g, 0.61 mmol), potassium carbonate (K_2CO_3) (2.79 g, 20.20 mmol) and *N*,*N*-dimethylacetamide (DMA) (30 mL) were added into a 100 mL RB flask and connected to a nitrogen purge line. With stirring at room temperature, tri-*tert*-butylphosphine (50 wt% in toluene, $P(t\text{-}Bu)_3$) (0.30 mL, 1.21 mmol) was added dropwisely into mixture. The reaction was refluxed for 8 h, and cooled down to room temperature. Excess amount of MC was added to dissolve product and the solution was filtered with silica gel/celite packed filter. The filtrate solution was concentrated and adsorbed into silica gel. The column purification was performed with a mixed solvent of MC and *n*-hexane (1:4). (1.55 g, Yield 71%) After that, recrystallization was proceeded with pure toluene. The recrystallized product was obtained by filtration, and further purified by train sublimation. A yellow powder was collected after all purification process.

^{1}H NMR (500 MHz, CD_2Cl_2) : δ 8.74 (s, 1H), 8.27 (s, 1H), 8.24 (d, *J* = 1.9 Hz, 1H), 8.18 (d, *J* = 7.2 Hz, 2H), 8.02 (d, *J* = 8.6 Hz, 1H), 8.01 (d, *J* = 8.5 Hz, 1H), 7.97 (d, *J* = 1.2 Hz, 1H), 7.79 (d, *J* = 2.2 Hz, 1H), 7.74 (d, *J* = 1.2 Hz, 1H), 7.69 (dd, *J* = 8.5, 2.0 Hz, 1H), 7.42 (dd, *J* =

8.6, 1.8 Hz, 1H), 7.27~7.30 (m, 4H), 7.24~7.26 (m, 4H), 7.05~7.07 (m, 4H), 7.02~7.04 (m, 4H), 1.58 (s, 9H), 1.51 (s, 9H), 1.32 (s, 18H), 1.30 (s, 18H).

^{13}C NMR (126 MHz, CD_2Cl_2) : δ 147.6, 147.1, 146.2, 145.7, 145.6, 144.8, 144.7, 143.5, 142.9, 139.1, 138.8, 137.4, 131.6, 126.5, 126.3, 125.8, 125.0, 125.0, 123.3, 122.4, 120.1, 119.1, 118.6, 116.5, 96.5, 36.3, 34.6, 34.5, 33.0, 32.1, 31.6.

LCMS (APCI) m/z : Found 1074.6523 [(M)$^+$]. Calculated For $C_{78}H_{82}N_4$: 1074.6539

EA (%): calculated for CHN: C, 87.1; H, 7.7; N, 5.2. Found: C, 86.7; H, 7.6; N, 5.2.

Synthesis of *N*^{2},*N*^{2},*N*^{7},*N*^{7}-tetrakis(4-(*tert*-butyl)phenyl)-9-(2,4-dibromo-5-(3,6-di-*tert*-butyl-9*H*-carbazol-9-yl)phenyl)-9*H*-carbazole-2,7-diamine

The synthetic procedure of *N*^{2},*N*^{2},*N*^{7},*N*^{7}-tetrakis(4-(*tert*-butyl)phenyl)-9-(2,4-dibromo-5-(3,6-di-*tert*-butyl-9*H*-carbazol-9-yl)phenyl)-9*H*-carbazole-2,7-diamine was almost the same as that of *N*^{3},*N*^{3},*N*^{6},*N*^{6}-tetrakis(4-(*tert*-butyl)phenyl)-9-(2,4-dibromo-5-(3,6-di-*tert*-butyl-9*H*-carbazol-9-yl)phenyl)-9*H*-carbazole-3,6-diamine except for starting material. *N*^{2},*N*^{2},*N*^{7},*N*^{7}-tetrakis(4-(*tert*-butyl)phenyl)-9*H*-carbazole-2,7-diamine (1.50 g, 2.07 mmol) was used as a starting material. After purification, a white powder was collected. (1.99 g, Yield 78%)

^{1}H NMR (300 MHz, DMSO-d_6) : δ 8.45 (s, 1H), 8.25 (s, 2H), 7.91 (d, J = 8.4 Hz, 2H), 7.87 (s, 1H), 7.37 (d, J = 8.4 Hz, 2H), 7.29 (d, J = 8.1 Hz, 8H), 6.96 (d, J = 7.8 Hz, 8H), 6.83 (d, J = 8.4 Hz, 2H), 6.73 (d, J = 9.0 Hz, 2H), 6.61 (s, 2H), 1.39 (s, 18H), 1.23 (s, 36H)

MS (APCI) m/z : Found 1235.25 [(M + H)$^+$]. Calculated For $C_{78}H_{84}Br_2N_4$: 1234.51

Synthesis of 13,16-di-*tert*-butyl-*N*^{1},*N*^{1},*N*^{6},*N*^{6}-tetrakis(4-(*tert*-butyl)phenyl)indolo[3,2,1-*jk*]indolo[1′,2′,3′:1,7]indolo[2,3-*b*]carbazole-1,6-diamine (1,6-tDPAtDIDCz)

The synthetic process and purification process were similar with those of 2,5-tDPAtDIDCz. As a starting material, *N*^{2},*N*^{2},*N*^{7},*N*^{7}-tetrakis(4-(*tert*-butyl)phenyl)-9-(2,4-dibromo-5-(3,6-di-

tert-butyl-9*H*-carbazol-9-yl)phenyl)-9*H*-carbazole-2,7-diamine (1.80 g, 1.45 mmol) was used instead of N^3,N^3,N^6,N^6-tetrakis(4-(*tert*-butyl)phenyl)-9-(2,4-dibromo-5-(3,6-di-*tert*-butyl-9*H*-carbazol-9-yl)phenyl)-9*H*-carbazole-3,6-diamine. Before recrystallization, a bright greenish powder was obtained. (0.65 g, Yield 42%)

^{1}H NMR (500 MHz, CD_2Cl_2) : δ 8.17 (d, *J* = 1.7 Hz, 1H), 8.10 (s, 1H), 7.96 (s, 1H), 7.91 (d, *J* = 8.5 Hz, 1H), 7.82 (s, 1H), 7.80 (d, *J* = 7.9 Hz, 1H), 7.78 (br. s, 1H), 7.65 (d, *J* = 8.5 Hz, 1H), 7.56 (dd, *J* = 8.5, 1.9 Hz, 1H), 7.43 (s, 1H), 7.41 (d, *J* = 8.6 Hz, 4H), 7.36 (d, *J* = 8.6 Hz, 4H), 7.21~7.38 (overlapped d+d, 4+4H), 7.16 (d, *J* = 7.5 Hz, 1H), 7.05 (d, *J* = 8.1 Hz, 1H), 1.58 (s, 9H), 1.47 (s, 9H), 1.40 (s, 18H), 1.30 (s, 18H).

^{13}C NMR (126 MHz, CD_2Cl_2) *δ* : 147.3, 146.7, 146.3, 145.4, 143.9, 140.0, 138.0, 137.5, 137.2, 130.6, 126.6, 125.6, 124.7, 124.4, 123.6, 122.9, 120.1, 118.7, 118.1, 116.2, 116.1, 113.3, 111.6, 106.9, 95.8, 36.2, 35.2, 34.7, 32.9, 32.0, 31.7, 30.1.

LCMS (APCI) m/z : Found 1074.6532 [(M)$^+$]. Calculated For $C_{78}H_{82}N_4$: 1074.6539

EA (%): calculated for CHN: C, 87.1; H, 7.7; N, 5.2. Found: C, 86.2 ; H, 7.7; N, 5.1.

Supplementary References

1. Cho YJ, Jeon SK, Lee S-S, Yu E, Lee JY. Donor interlocked molecular design for fluorescence-like narrow emission in deep blue thermally activated delayed fluorescent emitters. *Chem. Mater.* **28**, 5400-5405 (2016).
2. Ahn DH, et al. Highly efficient blue thermally activated delayed fluorescence emitters based on symmetrical and rigid oxygen-bridged boron acceptors. *Nat. Photon.* **13**, 540-546 (2019).
3. Sun S, et al. Efficient deep-blue thermally activated delayed fluorescence emitters based on diphenylsulfone-derivative acceptor. *Dyes Pigm.* **178**, 108367 (2020).
4. Lim H, Cheon HJ, Woo SJ, Kwon SK, Kim YH, Kim JJ. Highly Efficient Deep-Blue OLEDs using a TADF Emitter with a Narrow Emission Spectrum and High Horizontal Emitting Dipole Ratio. *Adv. Mater.* **32**, 2004083 (2020).
5. Tan HJ, et al. Deep-Blue OLEDs with Rec. 2020 Blue Gamut Compliance and EQE Over 22% Achieved by Conformation Engineering. *Adv. Mater.* **34**, 2200537 (2022).
6. Hatakeyama T, *et al.* Ultrapure blue thermally activated delayed fluorescence molecules: efficient HOMO–LUMO separation by the multiple resonance effect. *Adv. Mater.* **28**, 2777-2781 (2016).
7. Oda S, Kumano W, Hama T, Kawasumi R, Yoshiura K, Hatakeyama T. Carbazole-Based DABNA Analogues as Highly Efficient Thermally Activated Delayed Fluorescence Materials for Narrowband Organic Light-Emitting Diodes. *Angew. Chem.* **133**, 2918-2922 (2021).
8. Kondo Y, *et al.* Narrowband deep-blue organic light-emitting diode featuring an organoboron-based emitter. *Nat. Photonics* **13**, 678-682 (2019).
9. Oda S, Kawakami B, Horiuchi M, Yamasaki Y, Kawasumi R, Hatakeyama T. Ultra-

Narrowband Blue Multi-Resonance Thermally Activated Delayed Fluorescence Materials. *Adv. Sci.* **10**, 2205070 (2023).

10. Tanaka H, et al. Hypsochromic shift of multiple-resonance-induced thermally activated delayed fluorescence by oxygen atom incorporation. *Angew. Chem. Int. Ed.* **60**, 17910-17914 (2021).

11. Chan C-Y, et al. Two boron atoms versus one: high-performance deep-blue multi-resonance thermally activated delayed fluorescence emitters. *Chem. Comm.* **58**, 9377-9380 (2022).

12. Naveen KR, Lee H, Braveenth R, Yang KJ, Hwang SJ, Kwon JH. Deep blue diboron embedded multi-resonance thermally activated delayed fluorescence emitters for narrowband organic light emitting diodes. *Chem. Eng. J.* **432**, 134381 (2022).

13. Park IS, Yang M, Shibata H, Amanokura N, Yasuda T. Achieving Ultimate Narrowband and Ultrapure Blue Organic Light-Emitting Diodes Based on Polycyclo-Heteraborin Multi-Resonance Delayed-Fluorescence Emitters. *Adv. Mater.* **34**, 2107951 (2022).

14. Kim J, Chung W, Kim J, Lee J. Concentration quenching–resistant multiresonance thermally activated delayed fluorescence emitters. *Mater. Today Energy* **21**, 100792 (2021).

15. Wang Y, et al. A periphery cladding strategy to improve the performance of narrowband emitters, achieving deep-blue OLEDs with CIEy< 0.08 and external quantum efficiency approaching 20%. *Org. Electron.* **97**, 106275 (2021).

16. Liu G, Sasabe H, Kumada K, Arai H, Kido J. Nonbonding/Bonding Molecular Orbital Regulation of Nitrogen-Boron-Oxygen-embedded Blue/Green Multiresonant TADF Emitters with High Efficiency and Color Purity. *Chem. Eur. J.* **28**, e202201605 (2022).

17. Yang M, Park IS, Yasuda T. Full-color, narrowband, and high-efficiency electroluminescence from boron and carbazole embedded polycyclic heteroaromatics.

J. Am. Chem. Soc. **142**, 19468-19472 (2020).

18. Min H, Park IS, Yasuda T. cis-Quinacridone-Based Delayed Fluorescence Emitters: Seemingly Old but Renewed Functional Luminogens. *Angew. Chem.* **133**, 7721-7726 (2021).

19. Park IS, Min H, Yasuda T. Ultrafast Triplet–Singlet Exciton Interconversion in Narrowband Blue Organoboron Emitters Doped with Heavy Chalcogens. *Angew. Chem.* **134**, e202205684 (2022).

20. Bian J, et al. Ambipolar Self-Host Functionalization Accelerates Blue Multi-Resonance Thermally Activated Delayed Fluorescence with Internal Quantum Efficiency of 100%. *Adv. Mater.* **34**, 2110547 (2022).

21. Cheon HJ, Shin YS, Park NH, Lee JH, Kim YH. Boron-Based Multi-Resonance TADF Emitter with Suppressed Intermolecular Interaction and Isomer Formation for Efficient Pure Blue OLEDs. *Small* **18**, 2107574 (2022).

22. Yan ZP, et al. A Chiral Dual-Core Organoboron Structure Realizes Dual-Channel Enhanced Ultrapure Blue Emission and Highly Efficient Circularly Polarized Electroluminescence. *Adv. Mater.* **34**, 2204253 (2022).

23. Lee HL, Chung WJ, Lee JY. Narrowband and pure violet organic emitter with a full width at half maximum of 14 nm and y color coordinate of below 0.02. *Small* **16**, 1907569 (2020).

24. Patil VV, *et al.* Purely Spin-Vibronic Coupling Assisted Triplet to Singlet Up-Conversion for Real Deep Blue Organic Light-Emitting Diodes with Over 20% Efficiency and y Color Coordinate of 0.05. *Adv. Sci.* **8**, 2101137 (2021).

25. Lee HL, *et al.* Multiple-Resonance Extension and Spin-Vibronic-Coupling-Based Narrowband Blue Organic Fluorescence Emitters with Over 30% Quantum Efficiency. *Adv. Mater.* **34**, 2202464 (2022).

26. Lee N-J, *et al*. Highly efficient soluble materials for blue phosphorescent organic light–emitting diode. *Dyes Pigm.* **95**, 221-228 (2012).
